# Small molecule antagonist of the bone morphogenetic protein type I receptors suppresses growth and expression of Id1 and Id3 in lung cancer cells expressing Oct4 or nestin

**DOI:** 10.1186/1476-4598-12-129

**Published:** 2013-10-26

**Authors:** Elaine Langenfeld, Malik Deen, Emmanuel Zachariah, John Langenfeld

**Affiliations:** 1 Department of Surgery, Rutgers-Robert Wood Johnson Medical School, One Robert Wood Johnson Place, P.O. Box 19, New Brunswick, NJ 08903-0019, USA; 2 Department of Pathology, Rutgers-Robert Wood Johnson Medical School, One Robert Wood Johnson Place, P.O. Box 19, New Brunswick, NJ 08903-0019, USA; 3 Division of Thoracic Surgery, Rutgers-Robert Wood Johnson Medical School, One Robert Wood Johnson Place, P.O. Box 19, New Brunswick, NJ 08903-0019, USA; 4 Rutgers-Cancer Institute of New Jersey, New Brunswick, NJ 08903-0019, USA

**Keywords:** Oct4, Nestin, NueN, BMP, Antagonist, Id1, Id3, Cell growth, Cell death

## Abstract

**Background:**

Bone morphogenetic proteins (BMP) are embryonic morphogens that are aberrantly expressed in lung cancer. BMPs mediate cell fate decisions and self-renewal of stem cells, through transcription regulation of inhibitor of differentiation protein/DNA binding proteins (Id1-3). Inhibition of BMP signaling decreases growth and induces cell death of lung cancer cells lines by downregulating the expression of Id proteins. It is not known whether the BMP signaling cascade regulates growth and the expression of Id proteins of lung cancer cells expressing the stem cell markers Oct4 and/or nestin.

**Methods:**

Lung cancer cells expressing Oct4 or nestin were isolated from lung cancer cell lines by stably transfecting the Oct4 promoter or nestin promoter expression vectors that induce expression of the green fluorescent protein reporter.

**Results:**

Our studies suggest that lung cancer cells expressing Oct4 or nestin are different cell populations. Microarray and quantitative RT-PCR demonstrated that the expression of specific stem cell markers were different between isolated Oct4 and nestin cells. Both the Oct4 and nestin populations were more tumorigenic than controls but histologically they were quite different. The isolated Oct4 and nestin cells also responded differently to inhibition of BMP signaling. Blockade of BMP signaling with the BMP receptor antagonist DMH2 caused significant growth inhibition of both the Oct4 and nestin cell populations but only increased cell death in the nestin population. DMH2 also induced the expression of nestin in the Oct4 population but not in the nestin cells. We also show that BMP signaling is an important regulator of Id1 and Id3 in both the Oct4 and nestin cell populations. Furthermore, we show that NeuN is frequently expressed in NSCLC and provide evidence suggesting that Oct4 cells give rise to cancer cells expressing nestin and/or NeuN.

**Conclusion:**

These studies show that although biologically different, BMP signaling is growth promoting in cancer cells expressing Oct4 or nestin. Inhibition of BMP signaling decreases expression of Id proteins and suppresses growth of cancer cells expressing Oct4 or Nestin. Small molecule antagonists of the BMP type I receptors represent potential novel drugs to target the population of cancer cells expressing stem cell markers.

## Introduction

Lung cancer is the leading cause of cancer deaths in the world. More patients die from lung cancer than breast, colon, prostate, and kidney cancer combined. Approximately 85% of patients diagnosed with lung cancer will die from their disease. Lung cancers initially responding to chemotherapeutic agents will eventually develop resistance to therapy. The expression of stem markers Oct4 and/or nestin in cancer cells is associated with resistance to chemotherapeutic agents leading to treatment failures [1-5].

Cancer stem cells (CSC) have been defined as rare tumor cells with the capacity to self-renewal and initiate tumor growth in mouse xenografts that histologically recapitulate the primary tumor [6,7]. CSC are reported to be more resistant to chemotherapy agents and the induction of apoptosis compared to other populations of cells within the same tumor [8-11]. Self-renewal and chemotherapy resistance in cancer-initiating cells is mediate through the expression of inhibitor of differentiation/DNA binding proteins Id1 and Id3 [12-14].

CD44 and CD133 antigens are commonly used to isolate CSC from lung and other carcinomas [7,11,15-19]. Isolated CD44 and CD133 cancer cells also express stem cell regulators Oct4, Sox2, nanog, and nestin [11,20-23]. Oct4 is transiently expressed during early development in pluripotent stem cells and is required for self-renewal [24]. Nestin is a marker of neural progenitor cells and is frequently expressed in cancer cells of non-small cell lung carcinomas [21,25-27]. Although several studies have shown CD44 + and CD133 + cells initiate tumor growth at a significantly lower number of cells compared to the negative populations, CD44- and CD133- populations have also been reported be tumor initiating cells in some studies [17,28]. These studies suggest that further characterization of specific population of cancer cells may be needed.

Self-renewal is an essential mechanism required for stem cells to maintain long-term populating cells. Bone morphogenetic proteins 2 and 4 (BMP2/4) mediate self-renewal of embryonic stems by stimulating the expression of Id1 [29]. BMPs signal through transmembrane serine/kinases composed of type I (alk2, alk3, and alk6) and type II receptors. The BMP receptor complex phosphorylates smad-1/5, which then activates response elements on the Id1, Id2, and Id3 promoters [30,31]. Downregulation of type I BMP receptors with siRNA and selective small molecule antagonists decreases the phosphorylation of smad-1/5 causing a decrease in expression of Id, Id2, and Id3 in lung cancer cell lines [32]. The inhibition of BMP type I receptors also induces cell death and causes significant growth inhibition of lung cancer cell lines, which is mediated through the downregulation of Id proteins [32]. The role of the BMP signaling cascade regulating the expression of Id proteins and growth of cancer cells expressing Oct4 or nestin is not known.

We further delineate the heterogeneity of lung cancer by showing that Oct4, nestin, and Neun are expressed in lung cancer cell lines and primary lung tumors. We isolated from lung cancer cell lines, cells that express Oct4 or nestin. Our studies suggest that Oct4 and nestin expressing cancer cells are a different population of tumor-initiating cells. Inhibition of BMP signaling with the selective antagonist DMH2 caused a decrease in the expression of Id1/Id3 and induced significant growth inhibition of cancer cells expressing Oct4 or nestin. Blockade of BMP signaling with small molecule antagonists of the type I BMP receptors represents a potential means to regulate the growth of lung cancer cells expressing stem cell markers.

## Materials and methods

### Cell culture

The A549 and H1229 lung cancer cell lines were cultured in Dulbecco’s Modified Eagle’s medium (DMEM, Sigma Aldrich, St Louis, MO, USA) with 5% fetal bovine serum (FBS) [33]. The lung cancer cell lines H157, H727, U1752, and H358, and H865 were cultured in 90% RPMI and 10% FCS. The cell lines were obtained from ATCC and from Malcolm Brock, John Hopkins University.

### Expression vectors

The Oct4 promoter/EGFP plasmid vector was a gift from Wei Cui (Roslin Institute, Midiothian, UK [34]. The nestin promoter/EGFP was obtained from Rohan Humphrey (La Jolla, CA). The SM22 promoter/luciferase expression vector was obtained from Julian Solway (University of Chicago, Chicago IL) [35]. The SM22 promoter was cloned into the pAcGFP 1–1 expression vector at the XhoI/Hind III sites (Clontech, Palo Alto, CA). Cells were transfected using electroporation and then selected with neomycin. Control cells were transfected with pcDNA 3.1 vector (Invitrogen) expressing EGFP (Clontech).

### Human tumor samples

Human lung tumor tissue samples were obtained from the Rutgers Cancer Institute of New Jersey (CINJ) after approval by the institutional review board and ethics committee of the Rutgers Robert Wood Johnson Medical School. Protocol approval number, 0220013730. The review committee waived the need for consent since no patient identifiers were used.

### Cell death assay

Cells were plated in 6 well plates at 10^6^ cells per well and treated with 1 μM DMSO or 1 μM DMH2 for 48 hours. Adherent and floating cells were harvested and incubated with 0.1 mg/ml of ethidium bromide. Immediately after staining approximately 100 cells were counted and the percentage of cells that took up ethidium bromide was determined.

### Cell counts

Cells were plated into 6 well plates at 10^5^ cells per well and treated with 1 μM DMSO or 1 μM DMH2 for 7 days. The cells were detached with trypsin, stained with trypan blue, and the number of live cells counted using a hemacytometer.

### Immunoflourescent imaging

Immunofluorescent imaging was performed on both non-adherent and adherent cells as previously described [36]. Cells were trypsinized and immunofluorescent imaging performed or placed into cloning chambers (Nunc Lab-Tek, Rochester, NY). Briefly, cells were fixed with 3.7% formaldehyde, permeabilized with 0.5% Triton X, and blocked with 1% BSA/PBS. Cells were incubated with primary antibodies in 1X PBS/1% BSA at room temperature for one hour. Appropriate Alex Fluor 488, 568, or 647 (Invitrogen/Molecular Probes) conjugated secondary antibodies were used. The secondary antibody was added for one hour at room temperature. Controls were treated in the same manner but did not receive primary antibody. In all negative controls samples there was no fluorescent signal. Primary antibodies used were rabbit anti-Oct 4 (Santa Cruz, Santa Cruz, CA), rabbit anti-human nestin (Chemicon), mouse anti-human nestin (Chemicon), and mouse anti-NeuN (Chemicon, Temecula, CA). Fluorescent images were captured using a Nikon Eclipse TE 300 inverted epifluorescent microscope and a Cool Snap black and white digital camera. IP Lab imaging software was used to assign pseudo-color to each channel.

### Immunohistochemistry (IHC)

IHC was performed on formalin-fixed paraffin-embedded primary NSCLC and tumor xenografts in mice. Antibodies used were mouse anti-Oct4A (Cell Marque, Rocklin, CA), mouse anti-human nestin (Chemicon), mouse anti-NeuN (Chemicon, Temecula, CA), and mouse anti-smooth muscle actin (SMA) (clone 1A4) (Sigma, St. Louis, MI). IHC was performed on 5 μm tissue sections. Detection of Oct4 and NeuN on primary NSCLC used Tris-EDTA antigen retrieval using Vantana Benchmark XT automated IHC system. Seminoma was used as a positive control for Oct4 and normal brain for NeuN. For detection of nestin, NeuN, and SMA antigen retrieval was performed using Target Retrieval Solution (Dako Cytomation, Carpentaria, CA). On these samples, the Biomodule IHC Staining Kit (Invitrogen) was used as per the manufacture’s instructions. IHC on cell lines was performed by plating cells on glass cover slips, fixing in 4% paraformaldehyde for 10 minutes, incubating with primary Oct4 antibody for 1 hour, and using the biomodule IHC staining kit for detection.

### Quantification of gene expression

RNA was extracted using the RNeasy kit as per the manufacturer's instructions (Qiagen, Valencia, CA). DNAase was used to remove any DNA contamination. cDNA was generated using Advantage RT for PCR kit (BD BiosciencesClontech, Palo Alto, CA). Quantitative PCR was performed with the Stratagene Mx3005p real-time thermal cycler (Agilent Technologies) with predesigned and validated Taq-Man gene expression assays according to the manufacturer’s specifications (Life Technologies, Grand Island, NY). Reference numbers used are: GAPDH (Hs99999905_m1), actin (99999903_m1), ACVRL1 (alk1) (Hs00163543_m1), ACVR1A (alk2) (Hs00153836_m1), BMR1A (alk3) (Hs00831730_s1), BMPR1B (alk6) (Hs00176144_m1), Pou3f1 (Hs00538614_s1) CD133 (Hs01009250_m1), UBE2Q1 (Hs01079904_m1), Pank3 (Hs00388176_g1), and Sel1L (Hs01071406_m1), Negative control included all reagents except cDNA. Expression was normalized to GAPDH using the formula 2^∆ CT^.

SYBER Green was used to detect double-stranded DNA for the following primers. Nestin (F) 5′-GCC-CTG-ACC-ACT-CCA-GTT-TA-3′ (R) 5′-GGA-GTC-CTG-GAT-TTC-CTT-CC-3′, Sox-2 (F) 5′-CAT-CAC-CCA-CAG-CAA-ATG-AC-3′ (R) 5′-TGC-AAA-GCT-CCT-ACC-GTA-CC-3′. Oct4A specific primers were (F) 5′-TCC-CTT-CGC-AAG-CCC-TCA-T-3′ and (R) 5′-TGA-CGG-TGC-AGG-GCT-CCG-GGG-AGG-CCC-CAT-C-3′. Oct4 primers spanning the first intron were (set 2) (F) 5′-GAA-GCT-GGA-GAA-GGA-GAA-GC- 3′. (R) 5′-GCC-GGT-TAC-AGA-ACC-ACA-CT-3′. PCR products were run on a gel, cDNA purified, and sequenced (GENEWIZ, South Plainfield, NJ). Genomic contamination was examined by quantitative PCR of RNA samples. Negative control included all reagents except cDNA.

### Transient gene knockdown

Silencer Select Validated siRNA was used to knockdown expression of Oct4 (Life Technologies, Grand Island,NY), ID number S10871. Silencer Select Negative Control siRNA (4390843) was used to confirm specificity of knockdown. One million H1299 cells were transfected with 30 nM siRNA with the Nucleofector II (Amaxa Biosystems, Gaitherburg, MD) using the manufacture’s Nucleofector kit T. Optimization was performed using the enhanced green fluorescent reporter (EGFP) (Clontech) expressed in the pcDNA 3.1 vector (Invitrogen), which showed approximately 80% of the cells were transfected using this transfection protocol. Fourty-eight hours after transfection the expression of Oct4 expression was examined by quantitative PCR and Western blot analysis.

### Microarray

By FACS, 10^6^ cells expressing high levels of GFP were isolated from H1299 cells stably expressing the Oct4 promoter/GFP or Nestin promoter /GFP reporter vectors. After 24 hours total RNA was isolated using RNeasy Mini Kit as described by the manufacturer (Qiagen). DNAse treated RNA concentration was measured using NanoDrop 1000 spectrophotometer (Thermo Scientific) and the quality was analyzed with Bioanalyzer 2100 (Agilent). Spotted microarrays were used to identify differentially expressed genes between the Oct4/GFP and Nestin/GFP cells. After reverse transcription with SuperScript II, cDNA was transcribed and the samples were labeled with Cy3, and hybridized to human one array version 4.2 (HOA 4.2) DNA microarrays (Phalanx Biotech) containing 30,968 features probing for approximately 20,230 unique genes, according to standard procedures followed at the Functional Genomics of the Cancer Institute of New Jersey. Microarrays were scanned with the GenePix 4000B Scanner (Axon Instruments). The Gene Expression Omnibus (GEO) number for the microarray data is GSE49281.

### Flow cytometry

Flourescence activated cell sorting (FACS) analysis was performed using a Beckman Coulter Epics XL. Cell sorting was performed using MoFlo XDP cells sorter (Beckman, Coulter). Cell lines stably transfected with expression vectors were sorted for cells with high expression of GFP or no GFP expression. Post sorting FACS analysis was used to confirm expression. For FACS analysis, the primary antibody mouse anti-human CD44 (BP Parmingen, San Diego, CA) was added to cells on ice for 60 minutes. Secondary antibodies were added for 60 minutes on ice. Control cells were treated with secondary antibody only.

### Isolating cells from tumors

Tumor xenografts from mice were minced and treated with “digestion buffer” (10 ml HBSS, 50 mg collagenase powder, 200 μl 2.5% trypsin, 50 μl 1 M CaCl2, 50 μl DNAse). Fetal bovine serum (FBS) was added and samples were passed through a 100-micron filter. Cells were centrifuged and suspended in 3 ml of red blood cell lysis buffer (0.15 M ammonium chloride, 7 mM potassium bicarbonate, 0.09 mM tetrasodium EDTA) for 10 minutes. By FACS, the GFP (+) cells were then isolated.

### Western blot analysis

Total cellular protein was prepared using RIPA buffer containing a protease inhibitor cocktail and protein concentration was measured using the BCA assay as described [37]. In brief, protein was analyzed by SDS-PAGE, transferred to nitrocellulose (Schleicher and Schuell, Keene, NH). After blocking, the blots were incubated overnight at 4°C with the appropriate primary antibody in Tris-buffered saline with 1% Tween (TBST) and 5% non-fat milk. Secondary antibodies were applied for 1 hour at room temperature. Specific proteins were detected using the enhanced chemiluminescence system (Amersham, Arlington Heights, IL). The primary antibodies that were used were rabbit monoclonal anti-pSmad 1/5/8 (Cell signaling Technology, Danvers MA) rabbit anti-actin, an affinity isolated antigen specific antibody (Sigma, Saint Louis, MO), rabbit monoclonal anti-Id1, rabbit monoclonal anti-Id3 (Calbioreagents, San Mateo, CA), rabbit anti-Oct 4 (Santa Cruz, Santa Cruz, CA), mouse anti-human nestin (Chemicon), and mouse anti-NeuN (Chemicon, Temecula, CA).

### Differentiation of single cells

By FACS, the GFP (+) and GFP (−) cells were isolated from Oct4/GFP and Nestin/GFP cell lines and one-hundred cells placed into cloning chambers containing cell culture medium (Nunc Lab-Tek, Rochester, NY) [33]. Cells were cultured in regular culture media for approximately 14 days until colonies formed. Immunofluorescent imaging was then performed as described above.

### Statistical analysis

To compare two groups, a student t-test was used. Differences with P values ≤ .05 were considered statistically significant.

## Results

### Expression of Oct4 in lung cancer cell lines

**Figure 1:**
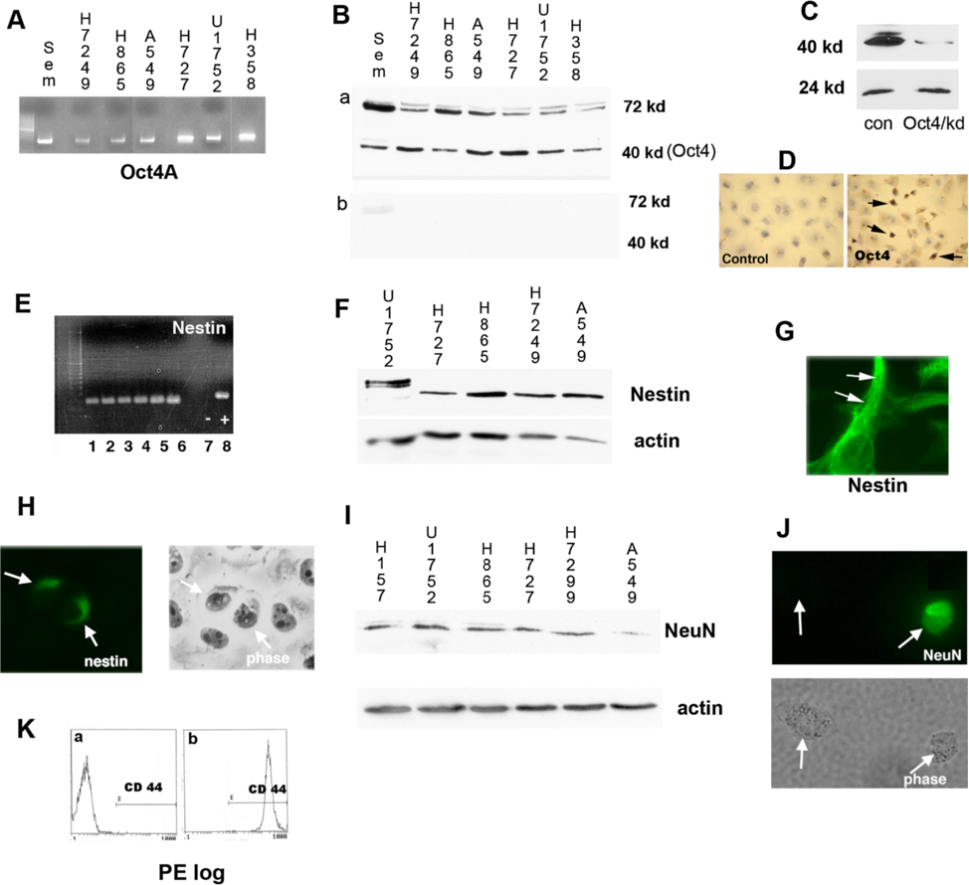
**Oct4A, nestin, and NeuN are expressed in lung cancer cell lines. (A)** PCR product of quantitative PCR on lung cancer cell lines using primer set 2 showing Oct4A expression. **(B-a)** Western blot analysis using antibody recognizing Oct4A (40 kd). **(B-b)** Western blot using only secondary antibody **(C)** Western blot analysis of Oct4 showing siRNA knockdown of Oct4. **(D)** Immunoflourscent imaging of H1299 cells showing nuclear expression of Oct4 (black arrows show positive cells). **(E)** RT-PCR demonstrating nestin expression in lung cancer cell lines shown in Figure 1A (lanes 1–6). Lane 8, is actin control, lane 7 negative control. **(F)** Western blot analysis showing nestin expression in lung cancer cell lines. **(G)** Immunoflourscent imaging of H1299 cells showing expression of nestin in cytoplasmic filaments (40x). **(H)** Immunoflourescent imaging and corresponding phase contrast image of H1299 cells showing nestin is expressed only in a subset of cells (20x). White arrows show immunopositive cells. **(I)** Western blot analysis showing the expression of NeuN in lung cancer cell lines. **(J)** Immunoflourescent imaging and corresponding phase contrast image showing nuclear expression of NeuN occurs in a subset of cells (20x). **(K)** FACS analysis for CD44 expression on H1299 cells. **(a)** control cells stained with phycoerythrin (PE) secondary antibody are without fluorescence. **(b)** Greater than 99% of cells stained with anti-CD44 and PE secondary demonstrated fluorescence.

Oct4 has two alternatively spliced variants that code the Oct4A and Oct4B isoforms. Oct4A regulates self-renewal of stem cells [38,39] and the function of Oct4B is not known. To evaluate Oct4A expression in our lung cancer cell lines, quantitative RT-PCR was performed. A seminoma (sem), was used as a positive control. A PCR product was amplified under 34 (29–34) cycles in all cell lines with no product in the negative control. Sequencing confirmed that the amplified product was Oct4A and not a pseudogene. To control for the presence of genomic contamination RNA samples were treated with DNase and PCR performed. This showed either no product or amplification at a high cycle number (38–39 cycles). In addition, to ensure cDNA was being amplified and not genomic DNA, primers recognizing exon 1 and exon 2 were used (primer set 2). Quantitative RT-PCR amplified the expected 420 base pair product at less than 34 cycles in all cells lines (Figure 1A).

Western blot analysis, using an antibody recognizing Oct4A, demonstrated bands at 72 kd and 40 kd in the seminoma and in all 6 lung cancer cell lines examined (Figure 1Ba). The expected size of Oct4A is approximately 40 kd. Western blot analysis using only the secondary antibody revealed a faint band at 72 kd in the seminoma (Figure 1Bb), suggesting that this could be a non-specific band. Western blot analysis showed that knockdown of Oct4 using siRNA targeting Oct4A showed a decrease in the 40 Kd band but not a non-specific 24 Kd band (Figure 1C). Quantitative PCR also showed a reduction in the expression of Oct4 following silencing of Oct4 with siRNA (Additional file 1: Figure S1). Immunohistochemistry demonstrated that Oct4 is expressed in the nucleus in approximately 16% of the cells within the cell lines (Figure 1D).

### Expression of nestin in lung cancer cell lines

By quantitative RT-PCR, nestin was expressed in all 6 lung cancer cell lines examined (Figure 1E). Amplification occurred under 34 cycles (26–34) in all of the cell lines. Sequencing of the PCR product confirmed that nestin was amplified. Western blot analysis for nestin showed strong expression in all the lung cancer cell lines examined (Figure 1F).

Immunofluorescent imaging of lung cancer cell lines showed that nestin is expressed in the cytoplasm filaments (Figure 1G). This was confirmed using both monoclonal and polyclonal anti-human Nestin antibodies. Nestin is expressed only in a subset of the cells (Figure 1H), which was approximately 20% within each cell line.

### NeuN expression in lung cancer cell lines

Since nestin is a marker of neural cells types, we examined whether lung cancer cells express NeuN (Neuronal Nuclei). NeuN is a protein detected in mature neurons [27]. Monoclonal antibodies detecting NeuN have shown that NeuN is not expressed in neural progenitors expressing nestin [40]. Western blot analysis using the monoclonal NeuN antibody showed an approximately 70 Kd band in all the lung cancer cell lines studied (Figure 1I). Immunoflourescent imaging showed nuclear expression of NeuN in the lung cancer cell lines (Figure 1J). Similar to Oct4 and Nestin expression, NeuN was expressed in approximately 15% of cells in each cell line (Figure 1J).

### CD44 expression in lung cancer cell lines

By FACS analysis, over 99% of A549 and H1299 cells expressed CD44 (Figure 1K and data not shown). Therefore, in A549 and H1299 lung cancer cell lines, CD44 does not appear to represent a specific population of cells.

### Oct4, nestin, and NeuN expression in primary NSCLC

**Figure 2:**
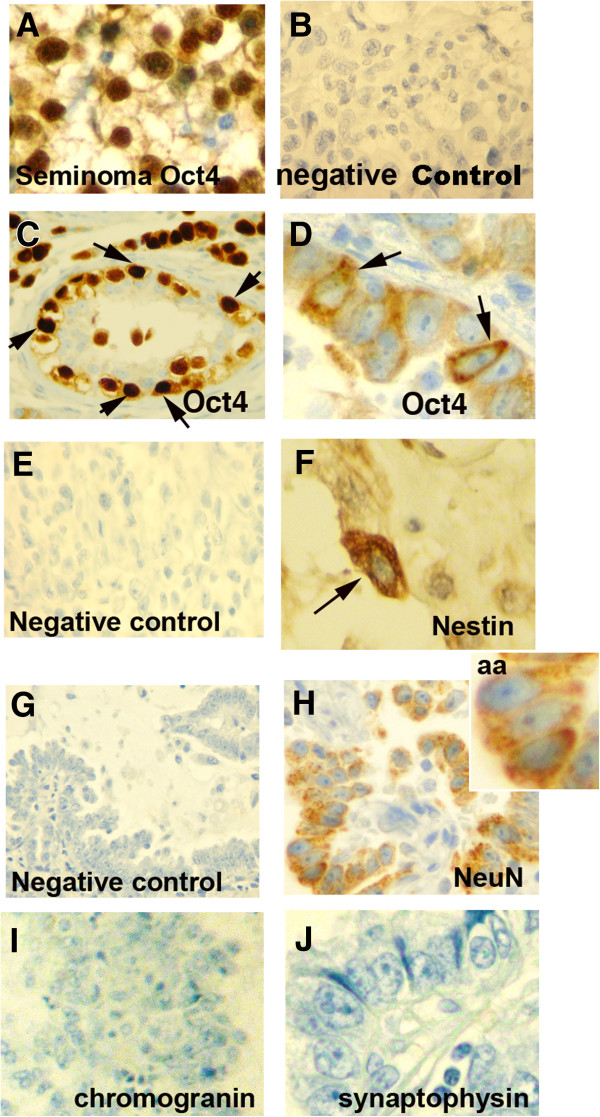
**Immunohistochemical (IHC) staining for Oct4, Nestin, and NeuN in primary NSCLC.** Shown are representative images. **(B, E, G)** Negative controls. Positive cells are stained brown. **(A)** Seminoma was used as positive control for Oct4. **(C)** shows nuclear staining in cancer cells for Oct4 and **(D)** shows cytoplasmic expression of Oct4. **(F-H)** Cancer cells expressing Nestin or NeuN expression. **(aa)** Magnified image of cells expressing NeuN. **(I-J)** Chromogranin and synaptophysin were not expressed in the NSCLC shown above.

To access whether the heterogeneity identified in the lung cancer cell lines occurs in primary lung cancer, we examine by immunohistochemistry (IHC) the expression of Oct4A, Nestin, and NeuN in NSCLC. Prior studies have reported that Oct4 and Nestin are expressed in NSCLC [20,41]. Using a monoclonal antibody recognizing Oct4A would, we showed nuclear expression of Oct4A in a seminoma (Figure 2A). Oct4A was expressed in 11 of the 12 NSCLC examined. Nuclear expression Oct4A was seen in one NSCLC (Figure 2C) while the cytoplasmic expression was demonstrated in 10 tumors (Figure 2D). Cell counts showed only 1-3% of the cancer cells expressed Oct4A. Nestin was expressed in frankly malignant cancer cells in 15 of 20 (75%) NSCLC (Figure 2F), which is consistent with prior reports [41]. The percentage of cancer cells expressing Nestin was from < 1% to 3% (Additional file 2: Table S1). Despite the NSCLC not having morphological features of neuroendocrine differentiation, 13 of 18 (72%) NSCLC expressed NeuN (Figure 2H). The percentage of cells expressing NeuN was significantly higher than that of Nestin (Additional file 2: Table S1). In comparison, the tumors that expressed thyroid transcription 1 (TTF-1) nearly all the cancer cells were immunopositive (Additional file 2: Table S1). To determine whether NeuN and/or Nestin immunopositive cells are of neuroendocrine or neural lineages, IHC for the neuroendocrine marker synaptophysin and chromogranin was performed. Only 1 of the 10 tumors positive NeuN and/or Nestin expressed synaptophysin (Figure 2I) and none expressed chromogranin (Figure 2J). This data suggest that cancer cells expressing NeuN or Nestin are of a neural lineage.

### Isolation of Oct4/GFP and nestin/GFP cells

**Figure 3:**
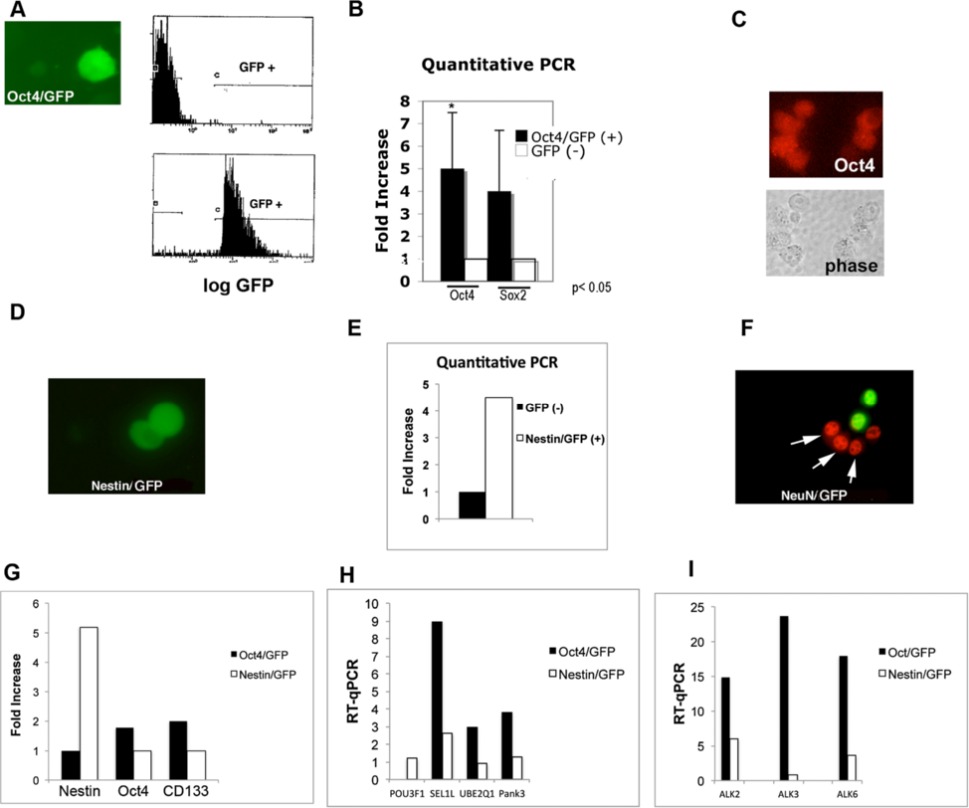
**Isolation of lung cancer cells expressing Oct4 or nestin.** Lung cancer cell lines were stably expressed with Oct4 or nestin promoters that drive GFP expression (Oct4/GFP and Nestin/GFP). **(A)** H1299 cell showing GFP expression after stable transfection with Oct4/GFP reporter. By FACS, GFP (+) cells were sorted from the Oct4/GFP cells and placed into cell culture. After 6 weeks cells were sorted for the GFP (+) and GFP (−) populations. **(A)** FACS analysis shows >99% purity of GFP (+) and GFP (−) cell populations. **(B)** Quantitative RT-PCR shows significantly higher expression of Oct4 and Sox2 in GFP (+) cells compared to GFP (−) cells (n = 3). **(C)** Immunoflourescent imaging showing Oct4/GFP (+) cells stain for Oct4. **(D)** The GFP (+) cells were sorted from H1299 cells stably expressing Nestin/GFP reporter and placed into cell culture. After approximately 8 weeks, the GFP (+) and GFP (−) cells were isolated by FACS. **(E)** Quantitative RT-PCR shows higher expression of nestin in GFP (+) cells compared to GFP (−) cells. **(F)** Dual immunoflourescent image shows NeuN (red) is not expressed in Nestin/GFP (+) (green) cells. **(G-I)** GFP (+) cells were isolated from Oct4/GFP and Nestin/GFP cells. Quantitative RT-PCR demonstrates differential expression of stem regulations and BMP type I receptors between Oct4/GFP and Nestin/GFP cells (n = 2).

A549, H1299, and U1752 cell lines were stably transfected with an expression vector containing promoters of Oct4 or nestin that regulates the expression of GFP. Human embryonic stem cells expressing this Oct4/GFP reporter in transgenic mice were shown to be pluripotent [34]. Somatic cells did not activate this exogenous Oct4 promoter construct [42]. Lung cancer cells expressing high levels of GFP were obtained in all 3 cell lines (Figure 3A). The GFP (+) cells were sorted by FACS and placed into cell culture. After approximately 6 weeks, the percentage of cells expressing GFP decreased to approximately 50% and 50% became GFP (−). By FACS, the GFP (+) and GFP (−) populations were again isolated to over 99% purity (Figure 3A). Quantitative RT-PCR showed that Oc4 and Sox2 expression was 4 to 5-fold higher in the GFP (+) cells in comparison to the GFP (−) cells (Figure 3B). Immunofluorescent imaging showed that the GFP (+) population expressed Oct4 (Figure 3C).

All 3 cell lines transfected with the Nestin/GFP reporter also showed strong GFP expression (Figure 3D). The GFP positive cells were isolated by FACS and plated into cell culture. After approximately 4 weeks the GFP + and GFP- cells were isolated. Quantitative PCR demonstrated a 4.5 fold higher expression of Nestin in the GFP + cells in comparison to the GFP (−) cells (Figure 3E). Dual immunofluorscent imaging showed that NeuN was not expressed in Nestin/GFP cells, suggesting that NeuN and Nestin represent different cell populations (Figure 3F).

### Expression profiles are different between the Oct4/GFP and Nestin/GFP cells

To examine differences in expression between the Oct4/GFP and Nestin/GFP cells, the GFP (+) cells were isolated to over 99% purity by FACS. Quantitative PCR demonstrated a five-fold higher expression of nestin in the Nestin/GFP cells compared to Oct4/GFP cells (Figure 3G). There was two-fold higher expression of Oct4 and CD133 in the Oct4/GFP cells compared to Nestin/GFP cells (Figure 3G). Microarray analysis showed that there were 603 genes that were differently expressed by >2 fold between GFP cells isolated from the Oct4/GFP and Nestin/GFP cells. By quantitative PCR, we examined selected genes that had a 4 fold or higher difference in expression and were related to cancer growth and/or stemness. Pou3f1, which is expressed in neural progenitors cells [43], was expressed in Nestin/GFP cells but not Oct4/GFP cells (Figure 3H). Sel1L, regulates self-fate decisions [44] and enhances tumor progression [45] was expressed over 3 fold more in Oct4/GFP cells than the Nestin/GFP cells (Figure 3H). UBE2q1 and Pank3 are regulators of cellular metabolism and enhance cell growth [46,47] were also confirmed to have a greater than 3 fold higher expression in the Oct4/GFP cells compared to Nestin/GFP cells (Figure 3H).

The level of expression of the BMP type I receptors differs between pluripotent stem cells and stem cell progenitors. Alk3 (BMPRIA) is expressed at much higher level in pluripotent stem cells compared to Alk6 (BMPRIB) [48]. Alk6 levels increase in some stem cell progenitors. To further assess differences between the Oct4/GFP and Nestin/GFP cells, the level of the BMP type I receptors alk2, alk3, and alk6 was examined by quantitative RT-PCR. GFP (+) isolated from Oct4/GFP cells showed a 26 fold higher expression of alk3 compared GFP (+) cells isolated from Nestin/GFP cells (Figure 3I). Alk2 and alk6 were expressed 2.5 and 5 fold higher respectively in the Oct4/GFP cells compared to the Nestin/GFP cells (Figure 3I).

### Oct4/GFP and Nestin/GFP cells are tumor initiating cells

**Figure 4:**
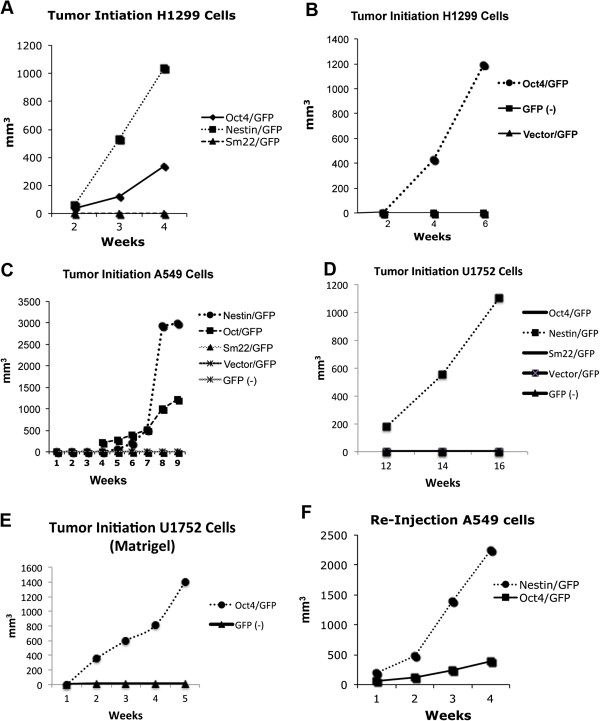
**Oct4/GFP and Nestin/GFP cells initiate tumor growth. (A-F)** Studies show differences in tumor initiation of different cell populations isolated from H1299, A549, and U1752 cell lines. **(A)** By FACS, 10^6^ GFP (+) cells were isolated from H1299 cells stably expressing Oct4/GFP, Nestin/GFP, or SM22/GFP expression vectors and injected subcutaneously into nude mice. **(B)** 10^5^ GFP (+) cells isolated form Vector/GFP and Oct4/GFP cells were injected into nude mice. GFP (−) cells were also isolated from Oct4/GFP (+) cells that became GFP (−) after being in cell culture for 6 weeks. **(C-D)** By FACS, 10^5^ GFP (+) were isolated from A549 and U1752 cells stably expressing Vector/GFP, Oct4/GFP, Nestin/GFP, SM22/GFP expression vectors and injected subcutaneously into nude mice. GFP (−) cells were isolated from Oct4/GFP cells. **(E)** 10^5^ GFP (+) and GFP (−) cells isolated from U1752 Oct4/GFP cells were injected into nude mice with Matrigel. These studies show that Oct4/GFP and Nestin/GFP cells are more tumorigenic than Vector/GFP, GFP (−), and SM22 cells, which did not form tumors at these cell concentrations. In addition, Nestin/GFP cells grow faster than Oct4/GFP cells. **(F)** 10^4^ GFP (+) cells isolated from Oct4/GFP and Nestin/GFP tumors re-established tumor after repeated injections into nude mice.

By FACS, the GFP + cells were isolated from the H1299, A549, and U1752 cells stably expressing the Oct4/GFP or the Nestin/GFP reporters. Controls were cells stably expressing GFP by a constitutively active CMV promoter (Vector/GFP) and Oct4/GFP + cells that lost GFP expression (GFP -) after isolation. Cell lines were also stably transfected with an expression vector containing the smooth muscle promoter, Sm22, that drives GFP expression. One million Sm22/GFP cells did not form tumors in mice after 4 months while 10^6^ Oct4/GFP and Nestin/GFP did (Figure 4A). One hundred thousand Vector/GFP (0 of 3) and GFP (−) (0 of 3) from the H1299, A549, and U1759 cell lines did not form tumors after 4 months, while the Nestin/GFP cells (3 of 3) and Oct4/GFP (2 of 3) developed tumors within 2 weeks (Figure 4B-D). One hundred thousand Sm22/GFP cells from A549 and U1759 cells did not form tumors after 4 months. In all three cell lines the Nestin/GFP grew faster than the Oct4/GFP cells.

One hundred thousand Oct4/GFP cells isolated from the U1752 cells did not initiate tumor growth (Figure 4D). Tumor initiation was slower in the U1752 cells taking 12 weeks for the Nestin/GFP cells to form a tumor. When 10^5^ Oct4/GFP cells derived from U1752 cells were co-injected with Matrigel a tumor formed within 3 weeks while the GFP (−) cells did not develop a tumor after 4 months. Therefore 3 of 3 Oct4/GFP and Nestin/GFP cells demonstrated ability to initiate tumor growth greater than Vector/GFP, Sm22/GFP, and GFP (−) controls.

Ten Oct4/GFP cells formed tumors compared the 10^4^ Vector/GFP cells (Additional file 3: Table S2). One-hundred thousand Oct4/GFP and Nestin/GFP cells isolated from the A549 and H1299 cell lines re-established tumor growth following re-injection into mice (Figure 4F and data not shown). The Nestin/GFP (+) cells again formed tumors that grew faster than the Oct4/GFP (+) cells (Figure 4F).

### Histologically the Oct4/GFP and nestin/GFP tumors are different

**Figure 5:**
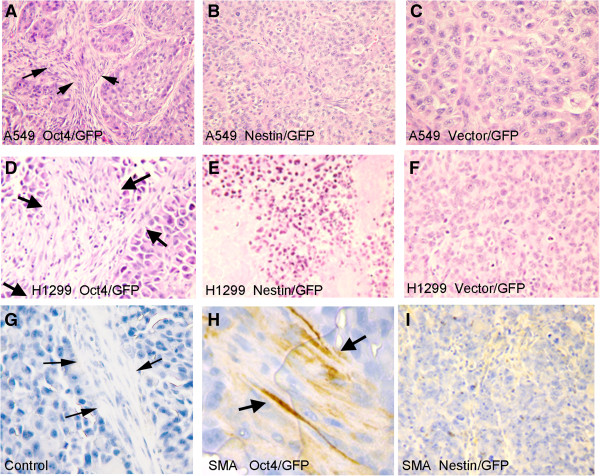
**Histologically the tumors formed from Oct4/GFP and Nestin/GFP cells are different**. Hematoxilin and Eosin (H & E) staining was performed on tumors formed from GFP (+) cells isolated from Oct4/GFP and Nestin/GFP of A549 and H1299 cell lines **(A-F)**. Tumors formed from 10^6^ Vector/GFP cells were used as a control. **(A,D)** Oct4/GFP cells formed more differentiated tumors with acini surrounded by large amounts of stromal tissue. Black arrows mark stromal tissue. **(B,E)** The Nestin/GFP and **(C,F)** Vector/GFP tumors were poorly differentiated with minimal stromal tissue. **(H)** IHC show that the stromal tissue found in Oct4/GFP tumors stain for smooth muscle actin (SMA). **(I)** Very little SMA was expressed in the Nestn/GFP tumors.

Hematoxylin and Eosin staining demonstrated that the Oct4/GFP and Nestin/GFP tumors recapitulated adenocarcinomas of the lung but the degree of differentiation was different. The Oct4/GFP (+) cells isolated from A549 and H1299 cells developed tumors that were more differentiated, forming glandular structures resembling acini (Figure 5A,D). The Nestin/GFP developed poorly differentiated tumors with no gland formation (Figure 5B,E). The Oct4/GFP tumors also had a large amount of stromal cells surrounding the acini, which stain for smooth muscle actin (SMA) demonstrating that they were either smooth muscle cells or myofibroblasts (Figure 5H). Tumors from the Nestin/GFP cells showed little expression of SMA (Figure 5I). One million unselected A549 and H1299 cells stably expressing GFP (Vector/GFP) developed poorly differentiated tumors with little stromal tissue (Figure 5C, F).

### BMP signaling in Oct4/GFP and Nestin/GFP cells

**Figure 6:**
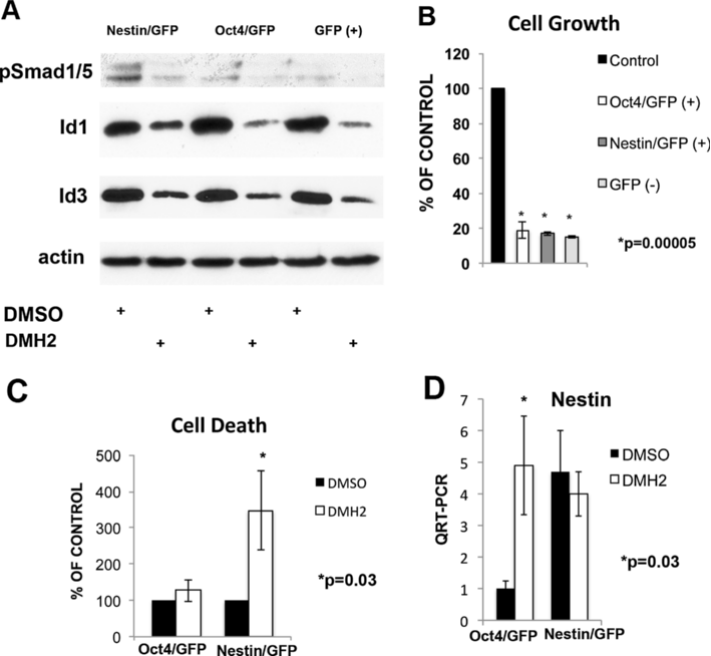
**Inhibition of BMP signaling decreases Id1 and Id3 expression and inhibits cell growth of Nestin/GFP, Oct4/GFP, and GFP (−) cells. (A)** Western blot analysis of the different cell populations isolated from H1299 cells treated with 1 μM DMH2 for 48 hours. DMH2 decreases pSmad 1/5 and its transcriptional target Id1 and Id3 in all cell populations. **(B)** Cells were treated with 1 μM DMH2 for 7 days and live cells counted. Data represents the mean of 3 independent experiments depicted as percent of vehicle control. **(C)** Oct4/GFP and Nestin/GFP cells were treated with 1 μM DMH2 for 48 hours and the percent cells that take up Ethidium bromide counted. Data represents the mean of 4 independent experiments shown as percent of vehicle control. Significant cell death occurred only in the Nestin/GFP cells. **(D)** Quantitative RT-PCR for nestin of Oct4/GFP and Nestin/GFP cells treated with 1 μM DMH2 for 48 hours. Data represents the mean of at least 3 independent experiments. Inhibition of BMP signaling induced nestin expression in Oct4/GFP cells but not Nestin/GFP cells.

The selective antagonist of the type I BMP receptor DMH2 causes significant growth suppression and a 3-fold increase in cell death of unselected H1299 and A549 cells, which involves the downregulation of Id1 and Id3 [32]. To assess whether BMP signaling cascade is active in cancer cells expressing stem cell markers, the Oct4/GFP, Nestin/GFP, and GFP (−) cells were treated with the DMH2. Western blot analysis demonstrated that DMH2 caused a significant reduction in the expression of the BMP transcription factor pSmad 1/5 and its direct downstream targets Id1 and Id3 in Oct4/GFP, Nestin/GFP, and GFP (−) cells (Figure 6A). DMH2 caused significant growth inhibition of Oct4/GFP, Nestin/GFP, and GFP (−) cells (6B). Inhibition of BMP signaling caused a significantly greater induction of cell death in the Nestin/GFP cells compared to the Oct4/GFP (Figure 6C). Since BMP signaling inhibits neural differentiation of embryonic stem cells [49,50], we examined whether DMH2 altered the expression of nestin in Oct4/GFP and/or Nestin/GFP cells. DMH2 induced a significant increase in the expression of nestin in the Oct4/GFP cells with a small decrease in expression in the Nestin/GFP cells (Figure 6D). DMH2 did not cause a significant change in the expression of Oct4 in either cell line (data not shown).

### Oct4/GFP cells gives rise to cells expressing nestin and NeuN

**Figure 7:**
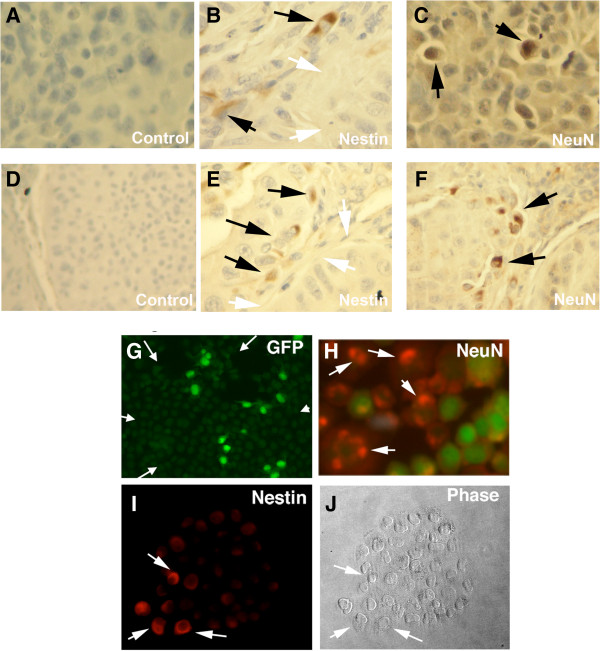
**Oct4/GFP (+) cells give rise to cells expressing nestin and NeuN.** 10^5^ GFP (+) cells were isolated from Oct4/GFP cells from A549 and H1299 cells and were injected subcutaneously into nude mice. IHC was performed on the tumors for nestin and NeuN from **(A-C)** A549 Oct4/GFP and **(D-F)** H1299 Oct4/GFP cells. **(A,D)** Represent negative controls. **(B,E)** Cancer cells expressing nestin (black arrow) were located along the periphery of the tumor acini (white arrow). **(C,F)** Cancer cells expressing NeuN (black arrows) were located toward the center of the tumor acini. **(G-J)** By FACS, GFP (+) cells were isolated from H1299 Oct4/GFP cells and single cells grown on glass cover slips for 2 weeks. Immunoflourscent imaging was performed on colonies for expression of GFP, Nestin, or NeuN. Shown are representative images of single colonies. **(G)** Immunflourescent image for GFP showing only a portion of cells in the colony express GFP. Arrows show the border of the colony. **(H)** Dual immunoflourescent for NeuN (red with white arrows) and GFP (green). **(I)** Immunoflourescent imaging for nestin (red) and **(J)** its corresponding phase contrast image. White arrows show cells expressing nestin.

The downregulation of Id1 in embryonic stem cells promotes differentiation [29]. The downregulation of Id1 and the induction of nestin in the Oct4/GFP cells following inhibition of BMP signaling suggested that the Oct4/GFP cells might undergo cellular differentiation. To assess differentiation, tumors formed from the Oct4/GFP cells were examined for the expression of nestin and NeuN. By IHC, approximately 3% of the cancer cells from the Oct4/GFP tumors expressed Nestin and NeuN (Figure 7). The nestin (+) cells localized to the periphery of the tumor acini and the NeuN (+) cells were identified in the center of the acini (Figure 7). Since the nestin (+) and NeuN (+) cells were identified in two different regions of the tumor suggests that they are two separate cell populations.

To further assess differentiation, colonies formed from a single Oct4/GFP cell were examined for the expression of nestin or NeuN. By FACS, the GFP (+) and GFP (−) cells were isolated from the H1299 Oct4/GFP cells and 100 cells plated onto glass cover slips. Microscopy confirmed that cells adhered to the cover slips as single cells. After two weeks colonies were examined by immunoflourescent imaging and the percentage of colonies staining for either NeuN or nestin were determined. The majority of the cells within each colony lost GFP expression (Figure 7G). All the colonies from the GFP (+) cells stained for NeuN and 96% stained for nestin (Figure 7 and Additional file 4: Tables S3 and Additional file 5: Table S4). The colonies from the GFP (−) cells stained for NeuN in 52% and nestin in 40% (Additional file 4: Tables S3 and Additional file 5: Table S4). Dual immunoflourescent imaging showed that cells expressing NeuN or nestin no longer expressed GFP (Figure 7 H-J). Only small percentage of the cells within each colony expressed either NeuN or nestin (Figure 7 H-J). Since only minority of the cells within a colony expressed NeuN or nestin, suggests that their expression occurs late in the colony formation. Together, these data support that Oct4/GFP cells give rise to cells that express NeuN or Nestin.

## Discussion

CD133+ and CD44+ cells are reported to represent “cancer stem cells” in lung carcinomas, which have also been shown to express Oct4 and/or nestin [11,20,21]. We provide evidence that lung cancer cells expressing Oct4 or nestin are different cell populations. The level of expression of nestin, BMP receptors, and other stem cell regulators are differentially expressed between the Oct4/GFP and Nestin/GFP cells. We also demonstrate biological differences between the Oct4/GFP and Nestin/GFP cells. The Nestin/GFP cells grew faster in nude mice than Oct4/GFP cells and form poorly differentiated tumors. The Oct4 cells formed more differentiated tumors and had a much large number of cells expressing smooth muscle actin. The response to BMP receptor antagonist also differed. DMH2 induced the expression of nestin in the Oct4/GFP + cells but not in the Nestin/GFP + cells. Inhibition of the BMP signaling cascade also caused more cell death in the Nestin/GFP cells compared to the Oct4/GFP cells.

We show that CD44 is expressed in nearly all cancer cells in our cell lines and CD133 is expressed in both Oct4 and nestin cell populations. Other reports have demonstrated that CD133 + cancer cells also express Oct4, nestin, nanog, and Sox2 [1,51]. The level of expression of Oct4 and/or nestin in cancer cells may induce specific survival mechanisms. Knockdown of nestin with siRNA decreases migration and invasiveness of pancreatic cancer cell lines [52]. Nestin regulates survival and self-renewal of neural stem cells [53]. Patients with NSCLC expressing nestin developed more metastasis and had a poorer survival [41]. Knockdown of Oct4 with siRNA in CD133 + lung cancer cells induced apoptosis, decreased tumorigenicity, and increased sensitivity to chemotherapy and radiation [20]. Our differentiation assays suggests that Oct4 cells give rise to cancer cells expressing nestin and NeuN. Further studies are needed to determine if a hierarchal organization occurs in “cancer stem cells” and examine the biology of other population of cells found within lung carcinomas.

BMP2 and BMP4 are highly conserved proteins required for development from insects to humans. BMP signaling is not active in adult lung tissue but is reactivated with inflammation and cancer [54,55]. BMP2 is highly overexpressed in 98% of NSCLC with little expression in paired normal lung tissue and benign lung tumors [55]. BMP-2 signaling is associated with poor prognosis and tumor progression [56,57]. BMP signaling has been shown to stimulate cancer growth, survival, migration, invasion, metastasis, and tumor angiogenesis of several different tumors [36,37,58-64]. We show that pharmacological blockade of BMP type I receptors causes significant growth inhibition of lung cancer cells expressing Oct4 or nestin. Inhibition of BMP signaling also caused significant growth inhibition and of non-selected cancer cells and GFP (−) cells, which were less tumorigenic. These data suggest that BMP antagonists affect the growth of more than just the Oct4 and nestin populations. Since cancer cells expressing stem cell makers represent only a small percentage of the cancer cells, therapeutically targeting the other cell populations is likely needed.

BMP receptor antagonists mediate growth inhibition of lung cancer cells by downregulating the expression of Id proteins [32]. BMP2/4 stimulates self-renewal of embryonic stem cells by inducing the expression of Id1 [29]. Studies have shown that Id1 mediates self-renewal of “cancer stem cells” and resistance to chemotherapy [12,13]. Within high grade gliomas, cancer cells with high Id expression (Id1-high) had a high self-renewal capacity [12]. Cancer cells with low expression of Id1 (Id1 low) were highly proliferative with little ability to self-renewal [12]. Inhibition of Id1 in Id1-high cells decreased self-renewal capacity and in Id1-low cells it decreased proliferation, suggesting that Id proteins have more than one biological function. Silencing of Id1 and Id3 together decreased self-renewal and increased sensitivity to chemotherapeutics of colon cancer-initiating cells [14]. We show that DMH2, a small molecule antagonist of the BMP type I receptors, effectively decreases Id1 and Id3 expression in lung cells expressing stem cell markers. Future studies are needed to determine whether BMP antagonists enhance the effectiveness of chemotherapeutics and decreases self-renewal of cancer cells expressing stem cell markers.

## Conclusion

These studies further delineate the heterogeneity of lung carcinomas. Our studies suggest that cancer cells expressing the stem cell markers Oct4 and nestin represent unique cell populations. We show that pharmacological blockade of the BMP/Id signaling cascade with small molecules targeting the type I BMP receptors causes significant growth inhibition of non-selected and cancer cells expressing stem cell markers. These studies provide further evidence that BMP receptor antagonists represent novel drugs for the treatment of cancer.

## Competing interests

A patent application was submitted for the use of BMP antagonists for the treatment of cancer. There have not been any royalties paid or anticipated in the near future regarding this work.

## Authors’ contributions

EL carried out the molecular biology studies and assisted interpretation of the data. MD carried out and analyzed immunohistochemistry studies. EZ performed and interpreted microarray studies. JL planned experimental design, interpreted all data, and drafted manuscript. All authors read and approved the final manuscript.

## Supplementary Material

Additional file 1: Figure S1 Quantitative RT-PCR showing siRNA decreases Oct4 expression in H1299 cells (n = 3). Click here for file

Additional file 2: Table S1 The percentage cancer cells within primary lung carcinomas expressing nestin, NeuN, or TTF-1 by immunohistochemistry. Click here for file

Additional file 3: Table S2 Tumor formation following injection of injection of 10,000, 100, and 10 cells from GFP (+) cells isolated from Oct4/GFP and Vector/GFP cells. Click here for file

Additional file 4: Table S3 By FACS, the GFP (+) and GFP (−) cells were sorted from H1299 Oct4/GFP cells and plated as single cells onto glass cover slips. After 2 weeks, colonies were stained for the expression of nestin and the number of positive colonies counted using immunoflourescent imaging. Click here for file

Additional file 5: Table S4 By FACS, the GFP (+) and GFP (−) cells were sorted from H1299 Oct4/GFP cells and plated as single cells onto glass cover slips. After 2 weeks, colonies were stained for the expression of NeuN and the number of positive colonies counted using immunoflourescent imaging. Click here for file
