# Supplementary figures and images for: Small molecule antagonist of the bone morphogenetic protein type I receptors suppresses growth and expression of Id1 and Id3 in lung cancer cells expressing Oct4 or nestin
Source: Mol Cancer. 2013 Oct 26;12:129. doi: 10.1186/1476-4598-12-129 (PMC4176118; doi:10.1186/1476-4598-12-129)

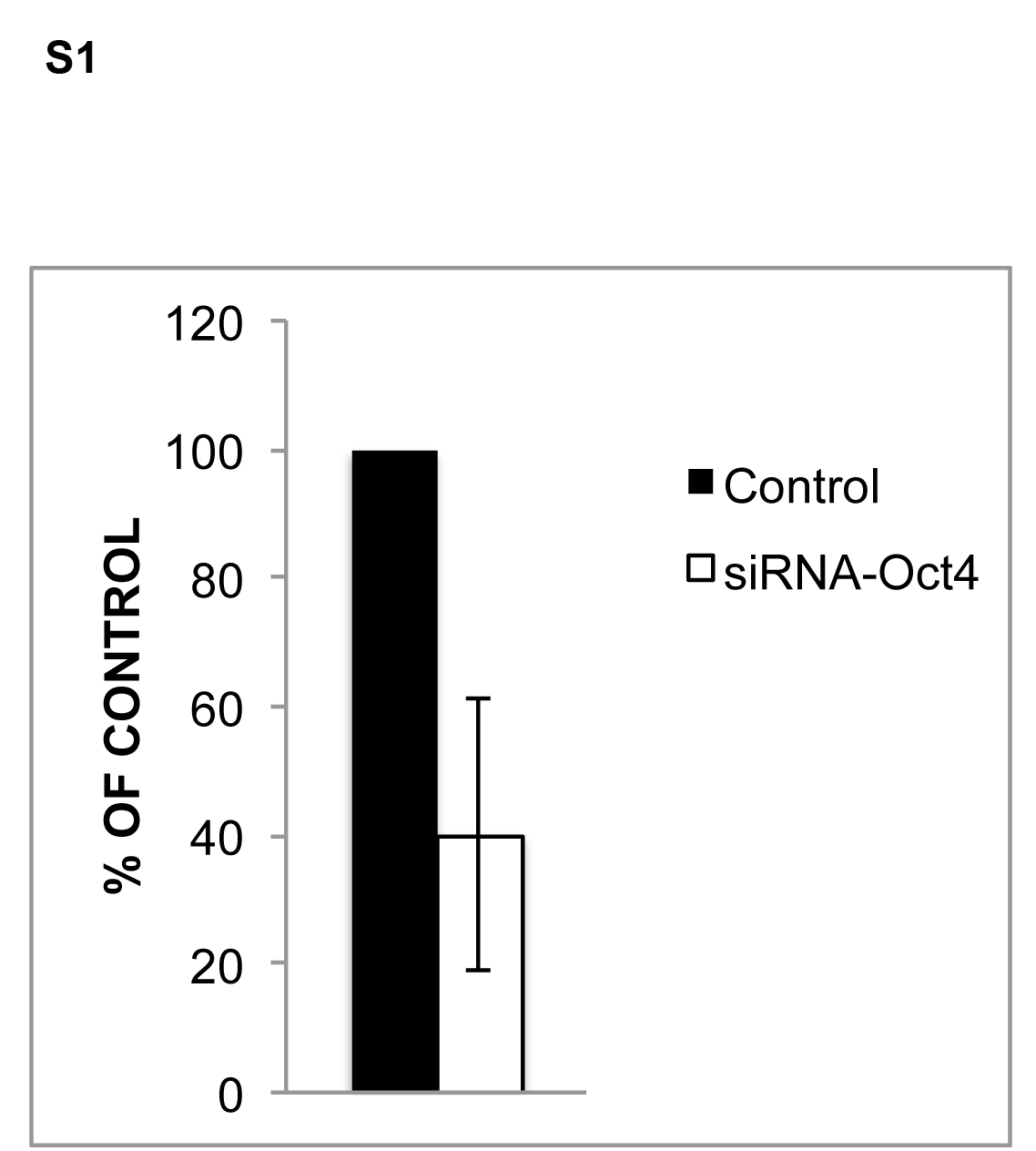

Supplement: Additional file 1: Figure S1 — Quantitative RT-PCR showing siRNA decreases Oct4 expression in H1299 cells (n = 3). [file 1476-4598-12-129-S1.tiff]

Table S2: Tumor Formation


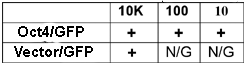


10,000, 100, and 10 cells were injected

into mice. (+) tumors formed. N/G =

no growth.

Supplement: Additional file 3: Table S2 — Tumor formation following injection of injection of 10,000, 100, and 10 cells from GFP (+) cells isolated from Oct4/GFP and Vector/GFP cells. [file 1476-4598-12-129-S3.doc]
