# Supplementary material for: Small molecule antagonist of the bone morphogenetic protein type I receptors suppresses growth and expression of Id1 and Id3 in lung cancer cells expressing Oct4 or nestin
Source: Mol Cancer. 2013 Oct 26;12:129. doi: 10.1186/1476-4598-12-129 (PMC4176118; doi:10.1186/1476-4598-12-129)
Supplement: Additional file 2: Table S1 — The percentage cancer cells within primary lung carcinomas expressing nestin, NeuN, or TTF-1 by immunohistochemistry. [file 1476-4598-12-129-S2.doc]

Table S1: Immunohistochemistry on primary lung adenocarcinomas

| **Case** | **Nestin** | **Neun** | **TTF-1** |
| --- | --- | --- | --- |
| NSCLC 1 | <1% | Negative | >95% |
| NSCLC 2 | 2% | 30% (cytoplasmic) | >95% |
| NSCLC 3 | <1% | 1% (cytoplasmic) | >95% |
| NSCLC 4 | 3% | 60% (cytoplasmic) | >95% |
| NSCLC 5 | <1% | 80% (cytoplasmic) | Positive ( not counted) |
| NSCLC 6 | 3% | 20% (cytoplasmic) | >95% |

Primary lung carcinomas were immunostained for the expression of nestin, NeuN, and TTF-1 and percentage of cancer cells staining were counted.
