# Supplementary material for: Small molecule antagonist of the bone morphogenetic protein type I receptors suppresses growth and expression of Id1 and Id3 in lung cancer cells expressing Oct4 or nestin
Source: Mol Cancer. 2013 Oct 26;12:129. doi: 10.1186/1476-4598-12-129 (PMC4176118; doi:10.1186/1476-4598-12-129)
Supplement: Additional file 4: Table S3 — By FACS, the GFP (+) and GFP (−) cells were sorted from H1299 Oct4/GFP cells and plated as single cells onto glass cover slips. After 2 weeks, colonies were stained for the expression of nestin and the number of positive colonies counted using immunoflourescent imaging. [file 1476-4598-12-129-S4.doc]

**Table S**3. Number of Colonies That Express Nestin

| **GFP (+) Cells** | | |  | **GFP (-) Cells** | | |
| --- | --- | --- | --- | --- | --- | --- |
| Experiment  Number | Immunopositive  Colonies | Number of colonies |  | Experiment  Number | Immunopositive  Colonies | Number of colonies |
| 1 | 10 | 10 |  | 1 | 10 | 35 |
| 2 | 30 | 32 |  | 2 | 15 | 31 |
| 3 | 25 | 26 |  | 3 | 16 | 36 |
|  |  |  |  |  |  |  |
| Total | 65 | 68 |  |  | 41 | 102 |

By FACS, the GFP (+) and GFP (-) cells were sorted from H1299 Oct4/GFP cells and plated as single cells onto glass cover slips. After 2 weeks, colonies were stained for the expression of nestin and the number of positive colonies counted using immunoflourescent imaging.
